# Veterinary World reviewer acknowledgment 2021

**DOI:** 10.14202/vetworld.2022.531-536

**Published:** 2022-03-07

**Authors:** A. V. Sherasiya, Nazir

**Affiliations:** Veterinary World, Star, Gulshan Park, NH-8A, Chandrapur Road, Wankaner - 363621, Dist. Morbi, Gujarat, India

## Contributing reviewers

Veterinary World editorial team would sincerely like to thank all of our reviewers who contributed to peer review for the journal in 2021.

**Table:** 

No.	Reviewer	Country	Reviewed articles
1	A. Chwalibog	Denmark	3
2	A. I. Paştiu	Romania	2
3	A. R. Silva	Brazil	2
4	A. Towhidi	Iran	1
6	Abdelfattah H Eladl	Egypt	1
7	Abel Villa-Mancera	Mexico	1
8	Abid Aslam Maan	Pakistan	1
9	Abolghasem Siyadatpanah	Iran	1
10	Adeyinka Jeremy Adedeji	Nigeria	1
11	Adolfo PazSilva	Spain	1
13	Ahmed Ali Mahmoud Saleh	Japan	2
14	Ahmed S. Abdoon	Egypt	3
15	Ahrar Khan	Pakistan	1
16	Ailian Geng	China	1
17	Alejandro Hidalgo	Chile	3
18	Alejo Menchaca	Uruguay	1
19	Alfredo Medrano	Mexico	2
20	Amira Taman	Egypt	1
21	Ammar Ahmad Khan	Pakistan	3
22	Amy C Murillo	USA	1
23	Andrea L. Looney	USA	1
24	Andrew A. Butler	USA	1
25	Anihouvi H. Anihouvi	Belgium	1
26	Ankit Magotra	India	1
27	Anna Stanicka	Poland	1
29	Antonello Santini	Italy	1
30	Antonio Bevilacqua	Italy	1
33	Archana Jain	India	3
35	Arda Sozcu	Turkey	2
36	Arda Yildirim	Turkey	2
37	Ariadna Flores Ortega	Mexico	2
39	Ariel C. Toloza	Argentina	1
40	Arockiasamy Arun Prince Milton	India	1
41	Ashok Dangi	India	1
43	Ashwani Kumar	India	2
44	Ayman Abdel-Aziz Swelum	Egypt	3
45	B. Hoffmann	Germany	1
46	Bahador Sarkari	Iran	2
47	Bambang Pontjo Priosoeryanto	Indonesia	1
48	Batoul Mohamed Izzularab	Egypt	2
49	Belgin Siriken	Turkey	2
50	Bernard Davoust	France	1
51	Breda Jakovac-Strajn	Slovenia	1
52	Brian D. Nielsen	USA	1
54	Buket Er Demirhan	Turkey	2
55	Bulent Ekiz	Turkey	1
56	C. Del Prete	Italy	2
57	C. M. Franco	Spain	1
58	Carlos Landaeta-Aqueveque	Chile	1
59	Chao-Nan Lin	Taiwan	1
60	Charleata A Carter	USA	2
61	Charoonluk Jirapattharasate	Thailand	1
62	Chongling Yan	China	1
63	Claude T Sabeta	South Africa	1
64	Claudio Genchi	Italy	2
65	Cord M. Brundage	USA	1
66	Dakshina Bisht	India	1
67	Dalia Hamza	Egypt	1
68	Danielle Bastos Araujo	Brazil	1
69	David Gleeson	Ireland	1
70	Davinson Chuka Anyogu	Nigeria	1
71	Deepa PM	India	1
72	Deepan Kishore	USA	2
73	Dhanush Krishna Balakrishnannair	India	2
74	Diana Riebold	Germany	1
76	Dmitriy V. Volokhov	USA	2
77	Domenico Galante	Italy	1
78	Domingo Fernández García	Spain	1
79	Dongwan Yoo	USA	1
80	Elina B. Reinoso	Argentina	1
81	Eloiza Teles Caldart	Brazil	1
82	Elshymaa A. Abdelnaby	Egypt	1
83	Emily A. Smith	USA	1
84	Enrica Zucca	Italy	1
85	Erika van Zyl	South Africa	2
86	Erma Safitri	Indonesia	2
87	Esther Kamau	Kenya	1
88	F. Pandolfi	UK	1
89	Fabrizio Bertelloni	Italy	3
90	Farida Khammar	Algeria	2
91	Fazul Nabi	Pakistan	1
92	Felipe da Silva Krawczak	Brazil	1
94	Francesca Maria Sarti	Italy	1
95	Francesco Mira	Italy	1
96	Françoise Leriche	France	1
97	Giuseppe Bertoni	Switzerland	2
98	Glayciane Costa Gois	Brazil	1
100	Gosia Zobel	New Zealand	1
102	Halit imik	Turkey	2
103	Hany Mhany Ragab AbdelLatif	Egypt	1
104	Harish Padh	India	1
105	Harvie P. Portugaliza	Philippines	3
106	Hasria Alang	Indonesia	2
107	Hassan Vatandoost	Iran	1
108	Hazem Shaheen	Egypt	1
109	Heba Ahmed Hussein Ahmed	Egypt	1
110	Heriberto Caballero-Ortega	Mexico	1
111	HuaJi Qiu	China	2
112	Huseyin Yilmaz	Turkey	1
113	Hussam Mohamed Mohamed Ibrahim	Egypt	1
114	Hussein Awad Hussein	Egypt	1
115	Hyunjin Shin	Korea	1
116	Ibrahim Adisa Raufu	Nigeria	1
117	Igor Yankin	USA	1
118	Indrasen Chauhan	India	3
119	Inga Stadaliene	Lithuania	2
120	Ioana Matei	Romania	1
121	Ipsita Mohanty	USA	5
122	Ismail Hakkı Tekiner	Turkey	2
123	J Efren Ramirez-Bribiesca	Mexico	1
124	J. P. Vico	Argentina	2
125	Jareerat Aiemsaard	Thailand	1
126	Jaruwan Khonmee	Thailand	1
127	Jerome Andonissamy	India	1
128	Jesus Alonso PantiMay	Mexico	1
129	Ji Bao	China	1
130	Jitender Dubey	USA	1
131	Josef illek	Czech Republic	3
132	Joseph G. Barnes	USA	1
133	Joshua Mbanga	Zimbabwe	1
134	Kalyan Sundar Das	India	1
135	Kannan Thandavan Arthanari	India	1
136	Kate Fenner	Australia	1
137	Katrin StrutzbergMinder	Germany	1
138	Kaushal Kishor Rajak	India	1
139	Kavous Solhjoo	Iran	1
140	Keita Iyori	Japan	1
141	kekungu puro	India	1
142	Keshore R. Bidasee	USA	1
143	Khan Sharun	India	1
144	Khawla Elati	Tunisia	1
145	Kosta Y. Mumcuoglu	Israel	1
146	Koushlesh Ranjan	India	1
147	Laxmi Narayan Sarangi	India	1
148	Lena Sizova	Russia	1
149	Lenin Lenin Aguirre Riofrio	Ecuador	1
150	Liben Chen	USA	1
151	Liga Kovalcuka	Latvia	2
152	Lin Jiang	China	1
153	Linous Munsimbwe	Zambia	2
154	Linsen Zan	China	1
156	Lucía Calleros	Uruguay	1
157	Luciana Catalina	Romania	1
159	Luis Nestor Apaza Ticona	Spain	1
160	Luis Tello	USA	1
162	Luisa De Martino	Italy	1
163	Luit Barkalita	Belgium	1
164	Lukasz Migdal	Poland	2
165	M. Duggan	UK	1
166	M. Teresa Sancho	Spain	1
167	M. Zamri Saad	Malaysia	3
168	Mabel Kamweli Aworh	Nigeria	2
169	Magdalena Zając	Poland	2
170	Mahmoud Mohey Elhaig	Egypt	1
171	Mahreen Ul Hassn	UK	1
172	Maja Josip Velhner	Serbia	2
173	Manuela Oliveira	Portugal	1
174	Marc Drillich	Austria	1
175	Maria Cristina Cozzi	Italy	1
176	Maria Fernanda Castro Burbarelli	Brazil	1
177	Maria Luisa Dettori	Italy	1
178	Mariana Malzoni Furtado	Brazil	1
179	Mariano E. Fernandez-Miyakawa	Argentina	1
180	Mário Manuel Dinis Ginja	Portugal	2
181	Marta Vascellari	Italy	1
182	Maryam Azizkhani	Iran	1
183	Maryam Dadar	Iran	1
184	Massimo Vignoli	Italy	1
185	Md Samun Sarker	Bangladesh	1
186	Md. Ahaduzzaman	Bangladesh	2
187	Md. Golzar Hossain	Bangladesh	1
188	Md. Sadequl Islam	Bangladesh	1
189	Mehmet Gultekin	Turkey	1
190	Michelle Moore	USA	1
191	Miguel Angel Barrera Silva	Mexico	1
192	Miguel Angel Garcia-Bereguiain	Ecuador	1
193	Miguel Quaresma	Portugal	2
194	Min Yue	China	2
195	Mirosław Mariusz Michalski	Poland	1
196	Mohamed Abdo Rizk	Japan	1
197	Mohamed E. Abd	Egypt	1
198	Mohamed G. Hedia	Egypt	1
199	Mohamed Hamed	Egypt	1
200	Mohamed S. Kamel	Egypt	1
204	Mohamed Salah Ayyat	Egypt	2
205	Mohammad Abdul Momin Siddique	Vietnam	1
206	Mohammad Borhan Alzghoul	Jordan	1
207	Mohammad Moniruzzaman	Bangladesh	1
210	Mohammad Zahangir Alam	Bangladesh	1
211	Mohammed A. E.	Egypt	3
212	Mohammed El-Houadfi	Morocco	1
213	Mohammed Fouad El Basuini	Egypt	2
214	Mohammed Manosur	Sudan	1
215	Mohammed S. ALhajj	Saudi Arabia	1
216	Mohan Singh Thakur	India	2
217	Monthon Lertcanawanichakul	Thailand	1
218	Mostafa F.N. Abushahba	USA	2
219	Moutaz Zarkawi	Syria	1
220	Muhammad Irfan-ur-Rehman Khan	Pakistan	2
221	Murat Sevik	Turkey	2
222	Nadeem Shabir	India	1
223	Natalia Kasica	Poland	1
224	Nathalia Maria del Pilar Correa Valencia	Colombia	2
225	Naveen Kondru	USA	2
226	Nazli Ercan	Turkey	1
227	Nesrein M. Hashem	Egypt	1
228	Olfat Anter Mahdy	Egypt	1
229	P.S. Erickson	USA	1
230	Padet Siriyasatien	Thailand	1
231	Paiboon Sithithaworn	Thailand	1
232	Pankaj Kumar	India	1
233	Paola Modesto	Italy	1
234	Paramanandham Krishnamoorthy	India	1
235	Patricia Escandón	Colombia	1
236	Peerapol Sukon	Thailand	1
237	Pengfei Li	China	1
238	Phillip J Rincker	USA	1
239	Pie Ntampaka	Rwanda	2
240	Pınar Peker Akalın	Turkey	2
241	Pınar Şanlıbaba	Turkey	2
242	Piotr Szweda	Poland	1
243	Pourouchottamane R	India	1
244	Prabhat Kumar Pankaj	India	1
245	Prapansak Srisapoome	Thailand	1
246	Qingping Wu	China	2
247	R.Umaya Suganthi	India	2
248	Rafiqul Islam	India	1
249	Rajender Kumar	India	1
250	Rajib Deb	India	1
251	Randa Ismail Alarousy	Egypt	2
252	Randall Lockwood	USA	1
253	Raquel Salazar Lugo	Venezuela	1
254	Reginaldo Nassar Ferreira	Brazil	1
255	Remil L. Galay	Philippines	1
256	Richard Churchchil	India	1
257	Rob Armstrong	USA	1
258	ROBERT L. SCHARFF	USA	2
259	Rolf karl Schuster	United Arab Emirates	1
260	Rosa Estela Quiroz Castaeda	Mexico	1
261	Ruben Barajas Cruz	Mexico	1
262	S. B. N. Rao	India	1
263	S. Gnat	Poland	2
264	S.K. Mondal	India	1
265	Saber Esmaeili	Iran	2
266	Sabrina Locatelli	France	1
267	Sabrina Sheila Greening	New Zealand	1
268	Saeed Alamian	Iran	1
269	Sahadeb Dey	India	1
270	Sahar Rihan Fadhel	Iraq	1
271	Salma Bessalah	Tunisia	2
272	Sana A M Elgayar	Egypt	1
273	Sandeep Ghatak	India	1
274	Sandeep Kaswan	India	1
275	Saurabh Kadyan	India	1
276	Sayed Haidar Abbas	China	1
277	Semíramis Guimarães	Brazil	1
278	Senol Celik	Turkey	2
279	Serkan Irfan Kose	Turkey	1
280	Shabnoor Iqbal	Pakistan	1
281	Shaimaa Ali Selim	Egypt	1
283	Shannon K French	Canada	1
284	Sheeva Bhattarai	Denmark	1
285	Shekhar Ramchandra Bhagwat	India	1
286	Shih-Ching Yen	Taiwan	1
287	Simona Martinotti	Italy	1
288	Sina Salajegheh Tazerji	Iran	2
289	Sintija Jonova	Latvia	1
290	Socorro Retana-Marquez	Mexico	4
291	Somwang Lekjing	Thailand	1
292	Song Gao	China	1
294	Sorin Daniel Dan	Romania	1
296	Spenser J. Babb-Biernacki	USA	1
297	Sreenu Makkena	India	1
298	Stephania Madrid Gaviria	Colombia	1
299	Süleyman Cilek	Turkey	4
300	sunday charles olaogun	Nigeria	1
301	Susana Astiz	Spain	2
302	Tanko Polycarp Nwunuji	Nigeria	1
303	Tapas Kumar Sar	India	1
304	Tariq Jamil	Germany	2
305	tequiero abuom okumu	Kenya	1
306	Tetiana BAKHUR	Ukraine	1
307	V. C. RAYULU	India	1
308	V. Di Marco	Italy	1
309	Vanessa S. Cruz	Brazil	5
310	Varij Nayan	India	2
311	Ved Prakash	India	1
312	Venkateswara rao Parimisetti	India	1
313	Vibuntita Chankitisakul	Thailand	1
314	Vijay J. Jadhav	India	1
315	Vijaya Kumar Anumolu	India	1
316	Vincenzo Tufarrelli	Italy	1
317	Vishal S Suthar	India	2
318	Wenliang Li	China	1
319	Widya Paramita Lokapirnasari	Indonesia	4
320	William Alberto CanFranco	Colombia	1
321	William J. Fielding	Bahamas	1
322	Win Surachetpong	Thailand	2
323	Wojciech Kozdruń	Poland	1
324	Wojciech Zygner	Poland	3
325	Xue Gao	China	1
326	Ya-Ching Lin	Taiwan	2
327	Yacine Titouche	Algeria	2
328	Yafeng Huang	China	1
329	Yaron Dekel	Israel	1
330	Yaser Hamadeh Tarazi	Jordan	1
331	Yasser M. Albadrany	Iraq	1
332	Yoon Kong	South Korea	1
333	Yosra AMdouni	Tunisia	2
334	Yosra Soltan	Egypt	1
335	Youssef A. Attia	Saudi Arabia	1
336	Yukita Sato	Japan	1
337	Yves Millemann	France	1
338	Zaven Karalyan	Armenia	2

